# Terms used to describe and define activities undertaken as a result of the medication review process: Do they require standardisation? A systematic review

**DOI:** 10.1007/s11096-022-01494-5

**Published:** 2022-11-21

**Authors:** Mohammed Alharthi, David Wright, Sion Scott, Jeanette Blacklock

**Affiliations:** 1 School of Allied Health Professions, University of Leicester, Leicester, UK; 2 College of Pharmacy, Taif University, Taif, Kingdom of Saudi Arabia

**Keywords:** Medication review, Standardisation, Systematic review, Taxonomy

## Abstract

**Background:**

Medication review (MR) is the systematic assessment of a patient’s medications by a healthcare practitioner. It is necessary to compare such MR interventions to rationalise differences between them and assess their impact. The development of an international taxonomy for terms used to describe activities undertaken within the MR process would facilitate quality of reporting, and the comparison of different interventions.

**Aim:**

To identify overarching and individual MR activity terms and definitions reported within studies where MR was the main intervention.

**Method:**

A systematic review of the literature was performed using search terms for ‘Intervention’ and ‘Outcome’. Papers with empirical data reporting and describing MR activities in English were included. The Mixed Method Appraisal Tool was used to assess research quality. Two researchers reviewed all included literature independently. Data extraction was performed using Cochrane Effective Practice and Organisation of Care to report study characteristics, and terms and definitions used to describe MR activities.

**Results:**

Twenty-one papers were included: eight quantitative non-randomised trials (38%), eight randomised controlled trials (38%), and five quantitative descriptive studies (24%). Overarching interventions such as ‘Clinical’, ‘Education’ and ‘Technical’ were identified with no standardised definitions. Terms used to describe the medication review activities, such as stop, start and change, varied with significant potential for ambiguity.

**Conclusion:**

The literature reports a variety of overlapping, ambiguous and undefined MR terms. As a result, comparing process evaluations from MR interventions may be difficult. A standardised taxonomy to describe, define and report MR activities is required.

**Supplementary Information:**

The online version contains supplementary material available at 10.1007/s11096-022-01494-5.

## Impact statements

- Ambiguous terminologies for the process of medication review combined with a lack of agreed definitions limit the ability to compare medication reviews between studies.
- The findings of this review will be used to underpin the development of a taxonomy to achieve international agreement on how medication review interventions are described.

## Introduction

Medication review (MR) is an integral part of the practice of many different healthcare professionals and is defined as a systematic assessment of a patient’s medicines with the goal of optimising medicine use and improving health outcomes [1]. According to the available information, Pharmaceutical Care Network Europe (PCNE) classifies the medication review into three levels: type 1 includes the medication history, type 2a includes medication history and patient interview, and type 2b includes medication history and clinical data [2]. Type 3, the most advanced level of review, includes medication history, patient interview, and clinical data. The goals of MR include minimising drug-related problems (DRPs) and recommending actions [1, 3]. Activities resulting from MR include medication discontinuation, addition, changing, dose increase, dose decrease and monitoring [4, 5]. So, whilst an international definition of the term ‘medication review’ has been developed, combined with the recognition that a number of activities result from it, there are no standardised, agreed terms or definitions to describe these ensuing activities.

For example, whilst one study may describe different MR activities as discontinue a drug, add a drug, change dose, change drug, change dosage form, and change timing [4], another may use cessation of drug, addition of drug, dose increase, dose decrease, replacement of drug, drug formulation change, schedule change and monitoring [5]. The terms treatment stop, treatment start, dose adaptation, therapy switch, and change timing of administration are also used to describe similar MR activities [6]. Researchers are increasingly interested in comprehending processes within interventions to enable them to better understand the mechanism of impact (‘how the provided intervention occurred’) [7]. This is achieved by looking at fidelity (‘whether the intervention delivered as intended’); dose (‘how much of the intervention is delivered to each participant’); and reach (‘the proportion of individuals who should have received the intervention’) [7]. Therefore, a standard definition of MR activities enables the terms fidelity, dose and reach to be compared accurately. However, the existence of locally developed MR activity classifications makes it difficult to compare the different process evaluations from various countries and settings. Furthermore, when different terms are used, it is frequently without an underpinning definition, the assumption being that the reader will automatically understand the activity from the term used. Also, this issue creates difficulties in finding the medication review activities in the evidence search and synthesis.

Whilst the term ‘discontinue’ may seem intuitive, it may be used to describe either active discontinuation of ongoing therapy or removal of a medicine from a medication list because it has not been supplied for an extended duration. This is different from actively stopping a medicine after discussion with a patient and agreeing on this course of action. For process evaluation purposes, it is potentially important that active discontinuation of ongoing therapy is differentiated from discontinuation largely for administrative purposes, namely, to update records. The first activity is far more likely to have an impact on the patient than the latter.

Frequently, authors further classify MR activities with overarching terms such as clinical, technical or education [8]. Similarly, these overarching terms have no agreed, standard definitions.

The lack of standardised terms and definitions for overarching and individual MR activities creates unnecessary difficulties for researchers wishing to compare their process data with similar interventions and studies. The findings from this study will be used to develop a standardised, consensus taxonomy for overarching and individual MR activities.

### Aim

The aim of this systematic review was to identify overarching and individual activity terms and definitions reported within studies, where MR was the main intervention, to inform the development of an international taxonomy.

## Method

This systematic review was reported according to Preferred Reporting Items for Systematic Review and Meta-Analysis Protocols (PRISMA) [9]. The systematic review protocol was developed and registered on the international database of Prospectively Registered Systematic Reviews (PROSPERO), with the registration number CRD 42,020,215,992. This systematic review was guided by Cochrane Effective Practice and Organisation of Care (EPOC). A scoping review had been performed before conducting this systematic review to identify search terms and data extraction tools, select the quality assessment tool, and review 10% of preliminary searches in order to establish inclusion and exclusion criteria; the full scoping review is attached in supplementary file 1. The search strategy is provided in supplementary file 2.

Search keywords were determined, with synonyms for medication review and for stop, start, monitor, decrease and increase identified.

Regarding participants in the included studies, no restrictions were given to age, gender, or medical condition of the patients or to healthcare professionals (HCPs). All healthcare settings were considered.

### Inclusion criteria

Papers meeting all of the following criteria were included:

- Studies that focused primarily on describing the nature of medication review interventions.
- Studies that describe medication review interventions: clinical, technical and education interventions.
- Medication reviews based on patients with multi-morbidity.
- Medication reviews conducted by any healthcare professional.
- Studies written in English.

### Exclusion criteria

Any paper having any of the following exclusion criteria was not included:

- Studies that focused on medication reviews within a single disease state such as those focusing on medication review of cardiovascular medications
- Conference proceedings
- Systematic reviews
- Abstracts
- Audits
- Posters
- Papers without empirical data such as protocols and editorials
- Pilot studies
- Grey literature studies

Databases searched were Embase (Excerpta Medica Database) (Ovid); Medline (Ovid); AMED (Alternative Medicine database) (EBSCO); PsycInfo (Psychological Information Database) (EBSCO); and CINAHL Complete (Cumulative Index to Nursing and Allied Health Literature) (EBSCO). Bibliographies of included studies were also searched. The search terms were carried out using Medical Subject Heading (MeSH) and other appropriate text words.

Search results for databases were exported into the reference manager Endnote × 9.3.3, where any duplication was identified and removed. Then, the remaining titles were exported to Microsoft Excel for titles, abstract and full-text screening. Search results were limited by two filters: publication type (journal) and the English language. An updated search was conducted in October 2021 to ensure that recent data had been included in this review.

All titles and abstracts were screened independently by two reviewers to check their eligibility against inclusion and exclusion criteria. Screening decisions were then compared between the two reviewers, and any discrepancy resolved by a discussion. A third independent reviewer was not required.

The number of screened titles, abstracts and full paper texts identified at each stage was recorded in a PRISMA diagram [9]. Inter-rater agreement was measured through Cohen’s Kappa coefficient for every stage of screening [10].

### Quality assessment

An independent, duplicate quality assessment of each study was undertaken (by MA and DW). All the included studies were assessed using the Mixed Method Appraisal Tool (MMAT) which enables appraisal of different types of study designs [11].

### Data extraction

In line with Cochrane Effective Practice and Organisation of Care (EPOC) [12], the following data were extracted from papers and abstracts by two independent researchers.

General characteristics of the included studies:

- Title, author(s)
- Year of study publication
- Study objective
- Study design
- Study setting
- Country of the study
  Characteristics of the participants:
- Target population (e.g., HCPs or patients)
- Participants’ sample size

Description of the main findings:

- All terms used to describe medication review interventions conducted by HCPs in different settings
- Description of medication review interventions

Data from eligible studies were then checked by two independent reviewers (MA and DW) to verify the accuracy and completeness of the included data. Any disagreement was resolved by a consensus between the reviewers.

### Data analysis

Medication review activities’ data were themed by the research team and collated to inform the development of a medication review activities’ taxonomy. All the medication review activities were extracted. The results were then analysed by narrative synthesis.

## Results

### Paper selection and description

**Fig. 1:**
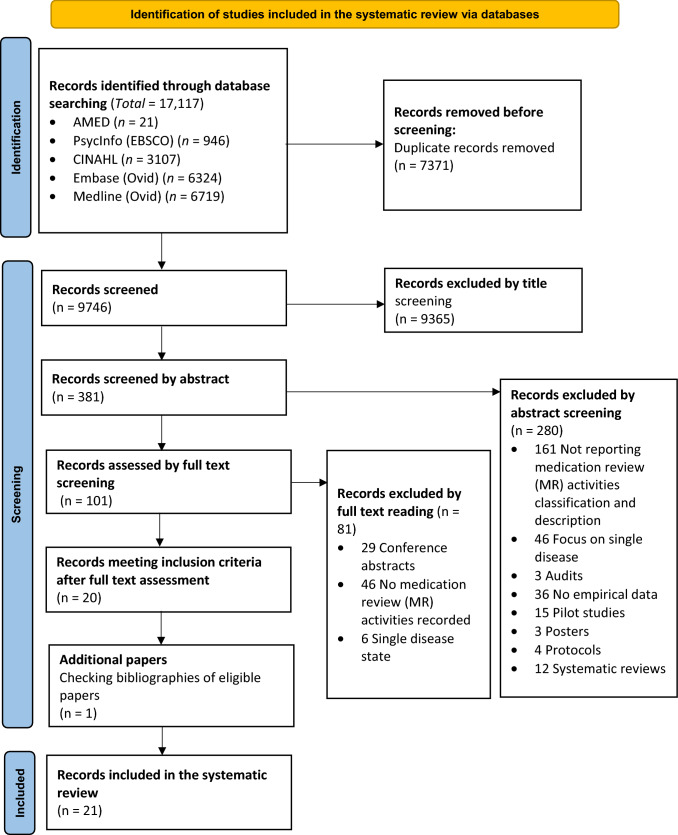
PRISMA flow diagram for literature review process [13]

The systematic search identified 17,117 citations, of which 21 met the inclusion criteria. The process of literature identification is summarised in a PRISMA flow diagram provided in Fig. 1. The level of agreement between independent reviewers at title, abstract and full text screening was 56.7%, 89.7% and 78%, respectively, with Kappa 0.18, 0.70 and 0.55, respectively.

Table 1 summarises the characteristics of the 21 papers that were included. Most of the studies were located in the UK, representing 28.5% of the total studies included (n = 6) [9, 14–18]; 23.8% from the Netherlands (n = 5) [5, 14, 18–20]; 19% from Australia (n = 4) [4, 21–23]; and smaller numbers (9.5%) from Sweden (n = 2) [24, 25]; 4.8% from Norway (n = 1) [26]; 4.8% from Belgium (n = 1) [5]; 4.8% from Canada (n = 1) [27]; and 4.8% from Jordan (n = 1) [28]. No specific study design was designated as this review was looking for papers for which MR activities and overarching activities had been described and defined. Accordingly, 38% of the papers were quantitative, non-randomised trials (n = 8) [5, 8, 14, 15, 20, 25, 29, 30]; 38% were randomised controlled trials (RCTs) (n = 8) [5, 16–19, 22, 23, 28]; and 24% were quantitative descriptions of medication review services (n = 5) [4, 21, 24, 26, 27]. The objectives of the studies included in this review varied and none of them described the nature of healthcare professionals’ activities, with the exception of two studies. Alldred et al. described them as stop medicine/stop drug, start medicine, dose increase/alter dose, dose reduction/alter dose, switch medicine/switch drug, alter formulation, alter timing, and test to monitor condition/test to monitor medicine [28] Petty et al. described them as stop drug, start drug, alter formulation dose, timing, switch drug, test request [15]. Papers ranged in publication date from 2002 to 2019.

**Table 1:** Summary of general characteristics of included studies

Title	Author, year, and country	Objective	Design	Setting	Target population	Sample size	Who delivered the intervention
Clinical medication review by a pharmacist of elderly people living in care homes: pharmacist interventions	Alldred et al. (2007) UK [29]	To describe the frequency and nature of pharmacist interventions following clinical medication reviews in elderly people living in nursing homes	Quantitative non—randomised	Care home	Residents aged + 65 years	331	Pharmacist
Clinical medication reviews in elderly patients with polypharmacy: a cross sectional study on drug-related problems in the Netherlands	Chau et al. (2015) Netherland [14]	To measure the presence of drug-related problems (DRPs) recognised through a clinical medication review and the level of implementation of proposed interventions in a large group of elderly patients with polypharmacy in community pharmacies	Quantitative non—randomised	Community pharmacies	Older patients aged + 65 years	3807	Community pharmacist
Drug-related problems (DRPs) and changes in drug utilisation after medication reviews in homes in Oslo, Norway	Fog et al. (2017) Norway [26]	Defining DRPs discovered throughout medication reviews (MRs) in nursing homes in Oslo, Norway	Quantitative descriptive	Nursing homes	Long term care patients	2465	Physician, nurse, clinical pharmacist
An evaluation of medication review reports across different settings	Christopher et al. (2012) Australia [4]	To contrast medication review reports completed by pharmacists working outside of a medical centre to those completed by an integrated practice pharmacist	Quantitative descriptive	A primary care medical centre	Medical centre patients	415	Pharmacist
Medication review by a clinical pharmacist at the transfer point from ICU to ward: a randomised controlled trial	Heselmans et al. (2015) Belgium [5]	To assess the impact of assigning a clinical pharmacist to the transfer process from intensive care unit to wards on the number and severity of drug-related problems	Randomised controlled trial (RCT)	Hospital	Intensive care unit patients (ICU)	1363	Pharmacist
Pharmacist-supported medication review training for general practitioners: feasibility and acceptability	Krska et al. (2006) UK [30]	To investigate the feasibility and acceptability of training in medication reviews provided by practice pharmacists for general practitioners (GPs)	Quantitative non—randomised	Primary care	General Practitioners	51	Pharmacist
Pharmacist-based medication review reduces potential drug-related problems in the elderly: the SMOG controlled trial	Vinks et al. (2009) Netherlands [18]	To see if a community pharmacist-led intervention reduces the number of potential DRPs in patients aged 65 and higher who are taking six or more medications concurrently	Randomised controlled trial (RCT)	Community pharmacies	Older patients aged + 65 years	100	Pharmacist
Effects of medication review on drug related problems in patients using automated drug dispensing system	Kwint et al. (2011) Netherlands [19]	To investigate the impact of a pharmacist-led medication review on drug-related problems (DRPs) in older patients receiving their medications through automated dispensing systems	Randomised controlled trial (RCT)	Primary care	Older patients	125	Pharmacist
The contribution of patient interviews to the identification of drug-related problems in home medication review	Kwint et al. (2012) Netherlands [20]	To ascertain the extent to which patient interviews aid in the identification of DRPs in terms of number, type, and clinical relevance	Quantitative non—randomised	Community pharmacies	Older patients aged + 65 years	155	Pharmacist
Completeness of medication review provided by community pharmacists	Kwint et al. (2013) Netherlands [5]	To investigate the completeness of DRPs in terms of quantity, category, and clinical significance as recognised by community pharmacists during home medication reviews (HMRs)	Quantitative non—randomised	Community pharmacies	Older patients aged + 65 years	155	Pharmacist
Performance of community pharmacists in providing clinical medication reviews	Laaksonen et al. (2010) UK [8]	To review the efficiency of trained community pharmacists in writing care plans and referrals for older patients as part of a patient outcome-focused medications management program	Quantitative non—randomised	Community pharmacies	Older patients aged + 65 years	461	Clinical pharmacist
Medication reviews in primary care in Sweden: importance of clinical pharmacists’ recommendations on drug-related problems	Modig et al. (2015) Sweden [24]	To assess the quality of clinical pharmacy service to primary care through structured medication review, with a focus on the therapeutic effect of clinical pharmacist suggestions	Quantitative descriptive	Primary care	Older patients	150	General practitioner, nurse, clinical pharmacist, and assistant nurse
Clinical medication review by a pharmacist of elderly patients on repeat medications in general practice: pharmacist interventions and review outcomes	Petty et al. (2002) UK [15]	To explore the nature of interventions carried out by a pharmacist during a clinical medication review in general practice	Quantitative non—randomised	General practice	Older patients aged + 65 years	1188	Pharmacist
General practitioner acceptance of medication review in Sydney nursing homes	Smith et al. (2002) Australia [21]	To perform a one-time medication review in nursing homes to identify medication issues and identify why suggestions made by the pharmacist conducting the review were not actioned by physicians	Quantitative descriptive	Nursing homes	Nursing home residents	202	Pharmacist
Clinical medication review by a pharmacist of elderly people living in care homes: randomised controlled trial	Zermansky et al. (2006) UK [16]	To assess the impact of a pharmacist-conducted clinical medication review versus standard care	Randomised controlled trial (RCT)	Care homes	Older patients aged + 65 years	331	Pharmacist
Pharmacists in humanitarian crisis settings: assessing the impact of pharmacist-delivered home medication management review service to Syrian refugees in Jordan	Al Alawaneh et al. (2019) Jordan [28]	To see how the home medication management review (HMMR) service affected the frequency of treatment-related problems (TRPs) between Syrian refugees living in Jordan	Randomised controlled trial (RCT)	Syrian refugees living in Jordan	Syrian refugees	106	Clinical pharmacist
Evaluation of collaborative medication reviews for high-risk older patients	Chan et al. (2018) Canada [27]	To quantify and identify the drug therapy issues identified and interventions implemented by pharmacists prior to and following the implementation of collaborative medication review for high-risk older adult patients	Quantitative descriptive	Hospital	Older patients	137	PharmacistFamily physician
Pharmacist-led medication review to identify medication-related problems in older people referred to an aged care assessment team	Elliott et al. (2012) Australia [22]	To compare three approaches to facilitating a pharmacist-led comprehensive medication review for people referred to an aged care assessment team (ACAT)	Randomised controlled trial (RCT)	Residential home	Older people	80	Clinical pharmacist
Effects of medication reviews on use of potentially inappropriate medications in elderly patients: a cross sectional study in Swedish primary care	Lenander (2018) Sweden [25]	To assess the impact of medication reviews on overall drug use and potentially inappropriate use in older patients, as well as to describe the frequency and categories of drug-related problems	Quantitative non—randomised	Nursing home	Older people	1720	Clinical pharmacist
Medication reviews in the community: results of randomised controlled trial	Sorensen (2004) Australia [23]	To assess the efficacy of a multidisciplinary service model that provides medication review to patients in the community who are at risk of medication misadventure	Randomised controlled trial (RCT)	Patients’ home	Polypharmacy patients	400	PhysicianPharmacist
Randomised controlled trial of clinical medication review by a pharmacist of elderly patients receiving repeat prescriptions in general practice	Zermansky et al. (2001) UK [17]	To determine whether a pharmacist can effectively review repeat prescriptions through consultations with elderly patients in general practice	Randomised controlled trial (RCT)	General practice	Older patients aged + 65 years	1188	Pharmacist

### Quality assessment

The quality assessment used the Mixed Method Appraisal Tool (MMAT) [11] for quantitative randomised controlled trials, quantitative non-randomised controlled trials, and quantitative descriptive studies. These are shown in supplementary file 3. Thirteen papers were allocated a score of 6 (62%) [5, 5, 8, 14, 16, 18–20, 22, 23, 25, 28, 29]; five papers a score of 7 (23.8%) [4, 21, 24, 26, 27]; and three papers a score of 5 (14.20%) [15, 23, 30].

### Descriptions and definitions of medication review interventions for clinical, technical and education interventions

Table 2 summarises terms used to describe the overarching activities which are ‘**clinical’, ‘technical’,** and **‘education’** interventions in the included studies.

**Table 2:** Summary of description of medication review activities for clinical, technical and education interventions

Author, year, and country	Overarching intervention categories					
	Clinical	Definitions for ‘clinical’ category	Technical	Definitions for ‘technical’ category	Education	Definition for education category
Alldred et al. (2007) [29]	Clinical	NA	Technical	NA	NA	NA
Chau et al. (2015) [14]	Clinical	NA	NA	NA	Provide education	NA
Christopher et al. (2012) [4]	NA	NA	NA	NA	Advice, suggest medication aid/advice/ monitor, advise administration techniques	NA
Heselmans et al. (2015) [5]	Clinical	NA	NA	NA	NA	NA
Krska et al. (2006) [30]	NA	NA	NA	NA	Lifestyle advice given to patient; advice given to patient about drug therapy	NA
Kwint et al. (2012) [20]	Clinical	NA	NA	NA	Education, counselling session	NA
Kwint et al. (2013) [5]	NA	NA	NA	NA	Education, counselling session	NA
Laaksonen et al. (2010) [8]	Clinical	NA	Clerical	NA	Provide counselling	NA
Modig et al. (2015) [24]	Clinical	NA	NA	NA	NA	NA
Petty et al. (2002) [15]	Clinical	The process where the HCP reviews the patient, the illness, and the drug treatment during the consultation	Technical	Altering quantities on prescription, deleting unused medicines, generic switch, adding dosage instructions	Compliance counselling	NA
Smith et al. (2002) [21]	Clinical	NA	NA	NA	NA	NA
Zermansky et al. (2006) [16]	Clinical	NA	Technical	NA	NA	NA
Al Alawaneh et al. (2018) [28]	Clinical	NA	NA	NA	Patient education, counselling on adherence	NA
Chan et al. (2018) [27]	Clinical	NA	NA	NA	NA	NA
Elliott et al. (2012) [22]	NA	NA	NA	NA	NA	NA
Sorensen et al. (2004) [23]	NA	NA	NA	NA	Provision of patient (or carer) education or information	NA

Eleven papers (53.3%) used term **‘clinical’** [5, 8, 14–16, 20, 21, 24, 27–29] and only one of them provided a definition for this term [15].

Four papers (19%) used the term **‘technical’** or **‘clerical’** [8, 15, 16, 29]and one of them provided a definition for this term [15].

Ten papers (47.6%) used the term **‘education’** or synonyms [4, 5, 8, 14, 15, 20, 22, 23, 28, 30] and none provided a definition for this term.

Table 2 shows that the authors agreed in their use of the ‘clinical’ term, whereas they described the technical intervention using two different terms, namely, ‘technical’ and ‘clerical.’ Furthermore, the education intervention was described in four different ways, namely, ‘educate,’ ‘advise,’ ‘aid,’ and ‘counsel.’

The authors used various terms to describe MR activities, as shown in Table 3. The term ‘stop’ has been described using four different words: ‘stop,’ ‘discontinue,’ ‘cease,’ and ‘withdraw.’ Three different words were used to describe the ‘start’ term: ‘start,’ ‘add,’ and ‘initiate.’ The term ‘increase’has been described by seven different words, namely, ‘increase’, ‘alter’, ‘adjust’, ‘change’, ‘adapt’, ‘rise’, and ‘titrate’. Dose decrease was described using seven different words: ‘reduce,’ ‘alter,’ ‘adjust,’ ‘change,’ ‘adapt,’ ‘decrease,’ and ‘titrate.’

**Table 3:** Summary of the terms used in previous studies to describe MR activity

Author, year, and country	Terms used to describe medication review activities							
	Terms used to describe ‘stop’ term	Terms used to describe ‘start’ term	Terms used to describe ‘dose increase’ term	Terms used to describe ‘dose decrease’ term	Terms used to describe ‘change’ term (for medication)	Terms used to describe ‘change’ term (for formulation)	Terms used to describe ‘change’ term (for timing)	Terms used to describe ‘monitor’ term
Alldred et al. (2007) UK [29]	Stop medicine/Stop drug	Start medicine	Dose increase/alter dose	Dose reduction/alter dose	Switch medicine/switch drug	Alter formulation	Alter timing	Conduct a test to monitor condition. Test to monitor medicine
Chau et al. (2015). Netherland [14]	Stop drug	Add drug	Adjust dose	Adjust dose	Switch drug	Switch dose form	Synchronise medication	Provide monitoring
Fog et al. (2017). Norway [26]	Stop the drug	Start new drug	Adjust the drug dose	Adjust the drug dose	Drug switch	NA	NA	Monitor the drug use
Christopher et al. (2012). Australia [4]	Discontinue drug	Add drug	Change dose	Change dose	Change drug	Change dosage form	Change timing, change schedule	Additional monitoring
Heselmans et al. (2015). Belgium [6]	Treatment stop	Treatment start	Dose adaptation	Dose adaptation	Therapy switch because interaction	NA	Change of moment of administration	NA
Krska et al. (2006). UK [30]	Drug stopped completely	New drug added	Dose changed	Dose changed	Drug changed to alternative	Formulation changed	NA	Monitoring done or ordered
Vinks et al. (2009). UK [18]	Cease a drug	Add drug	Change dose, dosage interval or frequency	Change dose, dosage interval or frequency	NA	Change dose form	NA	Laboratory tests
Kwint et al. (2011). Netherland [19]	Cessation of drug	Addition of drug	Dose change	Dose change	Replacement of drug	Drug formulation changed	Dose frequency/ schedule change	NA
Kwint et al. (2012). Netherland [20]	Cessation of drug	Addition of drug	Dose increase	Dose decrease	Replacement of drug	Drug formulation changed	Dose frequency/schedule change	Monitoring
Kwint et al. (2013). Netherland [5]	Cessation of drug	Addition of drug	Dose increase	Dose decrease	Replacement of drug	Drug formulation changed	Dose frequency/schedule change	Monitoring
Laaksonen (2010). UK [8]	Stop drug	Initiate therapy	Change dose or directions	Change dose or directions	Change drug or formulation within the same BNF subsection	Change drug or formulation within the same BNF subsection	NA	Start monitoring
Modig et al. (2015). Sweden [24]	Consider withdraw of drug therapy	Consider additional therapy/Initiation of therapy	Consider rise/increase dose	Consider reduce dosage	Consider change drug therapy	Consider change drug formulation	Consider change dosage regimen/dosage interval	Consider evaluation of therapy
Petty et al. (2002). UK [15]	Stop drug	Start drug (new indication)	Alter formulation, dose, timing	Alter formulation, dose, timing	Switch drug (same indication)	Alter formulation, dose, timing	Alter formulation, dose, timing	Test required
Smith et al. (2002). Australia [21]	Cease therapy	Initiate therapy	Increase dosage regimen	Reduce dosage regimen	NA	NA	NA	NA
Zermansky et al. (2006). UK [16]	Stop medicine	Start medicine	Alter dose	Alter dose	Switch	Alter formulation	Alter timing	Test required
Al Alaweneh et al. (2018) Jordan [28]	Ceasing drug	Adding drug	Changing therapeutic regimen (changing dose and/or frequency)	Changing therapeutic regimen (changing dose and/or frequency)	Changing drug	NA	NA	Monitoring (lab test or home-based monitoring)
Chan et al. (2018). Canada [27]	Discontinue a medication	Add or restart drug	Dose titration	Dose titration	Change medication or regimen	NA	Change medication or regimen	Monitor therapy
Elliott et al. (2012). Australia [22]	Stop drug	Add drug	Adjust dose	Adjust dose	Switch drug	Switch dose form	Adjust dose timing	Additional monitoring e.g., blood pressure, blood test
Lenander et al. (2018). Sweden [25]	Withdrawal of drug therapy	Initiation of drug therapy	Increased dose	Decreased dose	Change of drug therapy	Change drug formulation	Dose regimen adjustment	Evaluation of drug therapy
Sorensen et al. (2004). Australia [23]	Cease drug or trial withdrawal to confirm need for treatment	Add a drug	Change dose, dosage interval or frequency	Change dose, dosage interval or frequency	Change drug—substitute one for another	Change administration time, route, or dose form	Change administration time, route, or dose form	Monitoring, laboratory, monitoring observation and non-lab monitoring
Zermansky et al. (2001). UK [17]	Drug stopped	New drug started	Dose changed	Dose changed	Switched drug	Formulation changed	Frequency changed	NA

Table 3 demonstrates that medication changes, formulation changes, and timing changes are described in different ways. Words used to describe medication changes included ‘switch,’ ‘change’, ‘replace,’ and ‘alter’. The words ‘switch’, ‘change,’ and ‘alter’ were used to describe formulation changes. The words ‘change’, ‘synchronise,’ ‘alter,’ and ‘adjust’ were used to describe timing changes.

### Definitions of medication review terms

In seven of the included studies (33.3%) [4, 14, 15, 18, 23, 26, 27], the authors provided a description of some of the terms used for medication review as summarised in Table 4. The ‘Stop’ term was defined once (4.7%) [27], the ‘start’ term was defined four times (19%) [4, 15, 18, 27], and the ‘monitor’ term was defined three times (14.2%) [14, 23, 26].

**Table 4:** Summary of the definitions used in previous studies to define MR activity

Author, year, and country	‘Stop’ term	Definition used to describe ‘stop’ term	‘Start’ term	Definitions used to describe ‘start’ term	‘Monitor’ term	Definitions used to describe ‘monitor’ term
Chau et al. (2015) [14]	Stop drug	NA	Add drug	NA	Provide monitoring	Patient was monitored to decide whether the intervention was indicated
Fog et al. (2017) [26]	Stop the drug	NA	Start new drug	NA	Monitor the drug use	Monitoring of drug use
Christopher et al. (2012) [4]	Discontinue drug	NA	Add drug	Additional therapy required	Additional monitoring	NA
Vinks et al. (2009) [18]	Cease a drug	NA	Add drug	When another drug was started	Laboratory tests	NA
Petty et al. (2002) [15]	Stop drug	NA	Start drug (new indication)	Addition of drug for untreated indication	Test required	NA
Chan et al. (2018) [27]	Discontinue a medication	When drugs stopped during patient hospitalisation	Add or restart drug	When the patient needs additional drugs for an indication	Monitor therapy	NA
Sorensen (2004) [23]	Cease drug or trial withdrawal to confirm need for treatment	NA	Add a drug	NA	Monitor therapy	When drug therapy needs monitoring

Table 4 shows that some authors provided definitions for some of the terms such as ‘discontinue’, ‘add’ and ‘monitor’. However, the defined terms vary and were described by the authors without consensus.

## Discussion

### Main findings

This is the first systematic review to examine and propose standardisation of the way MR interventions are described. It highlights an absence of consistent terms used to define and describe such interventions. Terms used to describe instructions such as ‘alter’, ‘adapt’, ‘monitor’ lack sufficient detail to enable learning from and comparison of studies. There was limited uniformity with respect to overarching terms such as ‘clinical’ and ‘technical’, with the same activity being frequently classified differently between studies. There is a need for a consistent approach when describing medication review activities in studies.

### Strength and limitations

The search strategy employed was restricted to articles written in English. This risked rejecting potentially useful studies written in another language, reporting different HCPs’ activities.

The inclusion criteria used in this systematic review, together with the wide range of databases searched, have ensured that all the related studies from the largest possible number of articles initially identified were selected. However, having a greater number of initial studies to screen can result in less agreement. The large initial pool used in this systematic review may explain the lack of agreement between reviewers in the first stage of screening.

An extensive search was undertaken to gather all possible evidence regarding medication review activities, definitions and descriptions over time because there was no publication year restriction. The study designs ranged from randomised controlled trials to service evaluations in terms of quality. The Mixed Method Appraisal Tool (MMAT) showed that most studies reviewed were of high quality.

### Main discussion

The Mixed Method Appraisal Tool (MMAT) was chosen to review the included studies because no specific type of study was required, and it was expected that a variety of study types would be discovered when conducting the systematic review protocol.

It is worth noting at this point that, although they are not terms used to describe and define activities undertaken as a result of the medication review process, inconsistency was apparent to the authors in the use of the words ‘drug’, ‘medicine’, ‘medication’ and ‘treatment’. A ‘drug’ is defined as any chemical substance that affects the body's physiological process [31]. The term ‘treatment’ may refer to the use of an agent, procedure or regimen, such as surgery, drug, or exercise, in an attempt to cure or alleviate a medical issue. ‘Medicine' is defined as a formulated drug with a specific dose and dosage form used to prevent, diagnose, control, and treat disease [31]. It would seem that the latter is more appropriate. Consensus is required but it is outside the scope of this study.

The term ‘deprescribing’ first emerged in 2003 and there is a growing body of literature using it [32]. However, this systematic review showed that, despite being consistent with deprescribing, all papers researched used alternative terms under the overarching term ‘deprescribing’. This may be due to it being a relatively new term but also because it better describes the purpose rather than the activity—it is not a specific instruction. It can mean discontinuation, dose reduction or, potentially, dose increase if another medicine is up titrated to enable a second medicine to be stopped altogether. As such, it can be considered to be a generic term. However, there is growing literature on ‘deprescribing’ as an activity itself [33]. The medication review is a holistic process; the process of deprescribing includes reviewing all medications; identifying those that should be discontinued, substituted, or reduced; co-planning a deprescribing regimen with the patient; and regularly monitoring and supporting the patient [34].

The actions of stopping medication, changing dose, changing medication, formulation and monitoring are classed as clinical interventions. A clinical intervention is described as an activity performed to improve, maintain or assess the health of a person in a clinical situation. Altering the dose timing and changing from a branded to a generic version of a medication was defined as a technical intervention by some authors and as a clinical intervention by others. The classification disparity between authors of these two activities presents difficulties for researchers when comparing process evaluation studies. For example, Heselmans et al. [6] classified the medication timing change as a technical intervention [5], whilst the same activity was classified as a clinical intervention by Modig et al. [24]. Likewise, the medication formulation change is classified as a clinical intervention by Alldred et al. [29] and as a technical intervention by Chau et al. [14]. This inconsistency between authors in the use of overarching intervention categories indicates that there is a need to create a taxonomy to classify each of the conducted activities under the appropriate category which will enable meaningful comparison between process evaluations and help identify the mechanism of impact.

Various terms were used by different authors to indicate the same activity. For example, the terms ‘increase dose’ and ‘alter dose’ were used to describe one activity [29]. Another author applied the term ‘change dose’ to denote the dosage interval or frequency [18].

The act of stopping medication was described with different words in the included studies. The words ‘discontinue’, ‘cease’ and ‘stop’ suggest a permanent change whereas ‘suspend’ suggests a temporary change to medication.

The act of starting medication was described as ‘start’ and ‘restart’. It is crucial to separate the term ‘restart’ from ‘start’ because the former describes a medication deprescribing attempt that needs to be reversed.

The act of increasing the medication dose was described as ‘change dose’, ‘alter dose’, or ‘adjust dose’. Such terms do not specify whether the healthcare professional needs to increase the dose to improve medication effectiveness or decrease the medication dose to reduce side effects. Such terms give no indication of the precise instruction to be carried out or the reason for it. These vague descriptions and instructions give no indication as to the problem regarding the medication or the exact activity to be carried out by the practitioner. Despite some authors providing definitions for medication review terms used in their papers, these terms varied between authors. Such ambiguities would be eliminated by the development of a specific and inclusive consensus taxonomy of standardised medication review terms.

## Conclusion

This systematic review identified gaps in previous literature regarding the terms used by healthcare professionals with respect to the medication review process. Most of the studies included had wide and varied term definitions which demonstrated that there is no consensus terminology used by all researchers. The findings from this review support the need to develop a consensus between all healthcare professionals and researchers to use unique and specific terminology that facilitates process evaluation comparison, consequently providing a better understanding of the mechanism of impact.

As an outcome of this systematic review, the next step should aim to develop a consensus among a group of experts in the field of medication review to explore their decisions and agree the specific terms through a convenient and transparent consensus methodology. This proposal will be implemented in a future study by the authors.

## Supplementary Information

Below is the link to the electronic supplementary material.

Supplementary file1 (PDF 292 KB)

Supplementary file2 (PDF 122 KB)

Supplementary file3 (PDF 148 KB)

## References

[1] Griese-Mammen N, Hersberger KE, Messerli M (2018). PCNE definition of medication review: reaching agreement. Int J Clin Pharm.

[2] Imfeld-Isenegger TL, Soares IB, Makovec UN (2020). Community pharmacist-led medication review procedures across Europe: characterization, implementation and remuneration. Res Soc Adm Pharm.

[3] Beuscart J, Pont LG, Thevelin S (2017). A systematic review of the outcomes reported in trials of medication review in older patients: the need for a core outcome set. Br J Clin Pharmacol.

[4] Freeman CR, Cottrell WN, Kyle G (2013). An evaluation of medication review reports across different settings. Int J Clin Pharm.

[5] Kwint HF, Faber A, Gussekloo J (2014). Completeness of medication reviews provided by community pharmacists. J Clin Pharm Ther.

[6] Heselmans A, van Krieken J, Cootjans S (2015). Medication review by a clinical pharmacist at the transfer point from ICU to ward: a randomized controlled trial. J Clin Pharm Ther.

[7] Moore GF, Audrey S, Barker M (2015). Process evaluation of complex interventions: medical research council guidance. BMJ.

[8] Laaksonen R, Duggan C, Bates I (2010). Performance of community pharmacists in providing clinical medication reviews. Ann Pharmacother.

[9] Moher D, Liberati A, Tetzlaff J (2009). Preferred reporting items for systematic reviews and meta-analyses: the PRISMA statement. PLoS Med.

[10] Cohen J (1968). Weighted kappa: nominal scale agreement provision for scaled disagreement or partial credit. Psychol Bull.

[11] Hong QN, Fàbregues S, Bartlett G (2018). The mixed methods appraisal tool (MMAT) version 2018 for information professionals and researchers. Educ Inf.

[12] Mowatt G, Grimshaw JM, Davis DA (2001). Getting evidence into practice: the work of the cochrane effective practice and organization of care group (EPOC). J Contin Educ Health Prof.

[13] Page MJ, McKenzie JE, Bossuyt PM (2021). The PRISMA 2020 statement: an updated guideline for reporting systematic reviews. Syst Rev.

[14] Chau SH, Jansen APD, van de Ven PM (2016). Clinical medication reviews in elderly patients with polypharmacy: a cross-sectional study on drug-related problems in the Netherlands. Int J Clin Pharm.

[15] Petty DR, Zermansky AG, Raynor DK (2002). Clinical medication review by a pharmacist of elderly patients on repeat medications in general practice: pharmacist interventions and review outcomes. Int J Pharm Pract.

[16] Zermansky AG, Alldred DP, Petty DR (2006). Clinical medication review by a pharmacist of elderly people living in care homes: randomised controlled trial. Age Ageing.

[17] Zermansky AG, Petty DR, Raynor DK (2001). Primary care prescriptions in general practice. BMJ.

[18] Vinks THAM, Egberts TCG, de Lange TM (2009). Pharmacist-based medication review reduces potential drug-related problems in the elderly: the SMOG controlled trial. Drugs Aging.

[19] Kwint HF, Faber A, Gussekloo J (2011). Effects of medication review on drug-related problems in patients using automated drug-dispensing systems: a pragmatic randomized controlled study. Drugs Aging.

[20] Kwint HF, Faber A, Gussekloo J (2012). The contribution of patient interviews to the identification of drug-related problems in home medication review. J Clin Pharm Ther.

[21] Smith MA, Simpson JM, Benrimoj SI (2002). General practitioner acceptance of medication review in Sydney nursing homes. J Pharm Pract Res.

[22] Elliott RA, Martinac G, Campbell S (2012). Pharmacist-led medication review to identify medication-related problems in older people referred to an aged care assessment team: a randomized comparative study. Drugs Aging.

[23] Sorensen L, Stokes JA, Purdie DM (2004). Medication reviews in the community: results of a randomized, controlled effectiveness trial. Br J Clin Pharmacol..

[24] Modig S, Holmdahl L, Bondesson Å (2016). Medication reviews in primary care in Sweden: importance of clinical pharmacists’ recommendations on drug-related problems. Int J Clin Pharm.

[25] Lenander C, Bondesson Å, Viberg N (2018). Effects of medication reviews on use of potentially inappropriate medications in elderly patients: a cross-sectional study in Swedish primary care. BMC Health Serv Res.

[26] Fog AF, Kvalvaag G, Engedal K (2017). Drug-related problems and changes in drug utilization after medication reviews in nursing homes in Oslo, Norway. Scand J Prim Health Care.

[27] Chan WWT, Dahri K, Partovi N (2018). Evaluation of collaborative medication reviews for high-risk older adults. Can J Hosp Pharm.

[28] Al Alawneh M, Nuaimi N, Basheti IA (2019). Pharmacists in humanitarian crisis settings: assessing the impact of pharmacist-delivered home medication management review service to Syrian refugees in Jordan. Res Soc Adm Pharm.

[29] Alldred DP, Zermansky AG, Petty DR (2010). Clinical medication review by a pharmacist of elderly people living in care homes: pharmacist interventions. Int J Pharm Pract.

[30] Krska J, Gill D, Hansford D (2006). Pharmacist-supported medication review training for general practitioners: feasibility and acceptability. Med Educ.

[31] Walsh CT, Schwartz-Bloom RD. Pharmacology: drug actions and reactions. CRC Press; 2004. https://www.taylorfrancis.com/books/ISBN:9780203005798. Accessed 3 July 2022

[32] Woodward MC (2003). Deprescribing: achieving better health outcomes for older people through reducing medications. J Pharm Pract Res.

[33] Reeve E, Gnjidic D, Long J (2015). A systematic review of the emerging definition of ‘deprescribing’ with network analysis: implications for future research and clinical practice. Br J Clin Pharmacol.

[34] Beer C, Hyde Z, Almeida OP (2011). Quality use of medicines and health outcomes among a cohort of community dwelling older men: an observational study. Br J Clin Pharmacol.

