# Supplementary material for: Terms used to describe and define activities undertaken as a result of the medication review process: Do they require standardisation? A systematic review
Source: Int J Clin Pharm. 2022 Nov 21;45(2):304–19. doi: 10.1007/s11096-022-01494-5 (PMC10147810; doi:10.1007/s11096-022-01494-5)
Supplement: Supplementary file 1 — Supplementary file1 (PDF 292 KB) [file 11096_2022_1494_MOESM1_ESM.pdf]

# Scoping review to develop systematic review protocol.

## 1.1. Aim

The aim of the scoping review is to develop the protocol of the systematic review to identify the terms had been used to describe and define medication review activities.

## 1.2. Objectives

1. To conduct a review of existing systematic reviews that reported on or investigated medication review interventions in order to identify databases that were used in those reviews and may be used in this systematic review.
2. To identify search terms according to Population, Intervention, Comparison and Outcome (PICO) framework (1).
3. To review the titles and 10% of abstracts and full-text articles in order to establish inclusion and exclusion criteria and to develop the procedure for this systematic review.
4. Identify the data extraction tool
5. To select the quality assessment tool.

## 1.3. Methods:

### 1.3.1. Process of database identification

Nine previously published systematic reviews reported or investigated medication reviews in different settings were reviewed to explore what databases had the researchers selected for their studies. However, the researcher collated all of these databases and selected what to use for the study with respect to the inclusion and exclusion criteria for this systematic review. A number of these databases were excluded as they did not fit our study. The databases were excluded if they contained abstracts only, if they were not written in the English language, if they were limited to recent articles and if they contained non peer reviewed papers.

However, for purposes of the scoping review the search conducted on the following:

- AMED
- Embase
- Medline

## 1.3.2. Keyword selection

Keywords were built, following review, according to the PICO framework (2), which had been selected for assisting the researcher in obtaining more precise and relevant results. PICO breaks down a research keywords into four parts: population (P), intervention (I), comparison (C), and outcome (O). Therefore, it improves the study question's precision and conceptual clarity. The researcher chose the keywords from the following two sources:

1. Through reading relevant papers that either investigated or reported the effects or the outcomes of the medication review in different settings. Using the information reviewed, the researcher studied the nature of the medication review activities undertaken and the language used in their interventions. At this stage the researcher identified the keywords related to the intervention (I) and initial words of the outcomes (O)
2. The researcher reviewed a very large number of care plans (370 in total) for pharmacists' independent prescribers in CHIPPS trial (as stated in Chapter 2) and examined the nature and language of the interventions that had been undertaken during the medication review activities. At this stage the researcher identified the synonyms for outcomes (O).

For clarification, the terms were started with:

**P:** There is no specific population we are looking for, so this will be general, and no keywords could be added. The researcher will not search for the medication review activities in care homes only but will search all terms in other settings that provided medication review activities by health care professionals to patients' medications.

**I:** Medication review, drug utilisation review.

**C:** There is no comparison so no specific keywords could be added.

**O:** Deprescribe, start, change, reduce, increase, monitor, educate.

Then, synonyms for outcomes were identified from the CHIPPS pharmaceutical care plan as in table 1.

**Table 1:** Summary of outcome (O) keywords identified, and their synonyms from the PCPs

| **Initial keywords for outcomes** | Stop | Start | Change | Reduce | Increase | Monitor | Educate |
|---|---|---|---|---|---|---|---|
| **Synonyms from PCPs** | Deprescribe Discontinue | Commence initiate | Switch Alter | Step down | Step up | Test | Train Advise |

| | | | | | | | |
|---|---|---|---|---|---|---|---|
| | Hold | | | | | | |

Then, after an initial search, the researcher and the primary supervisor reviewed all of the titles aiming to exclude irrelevant words from the titles in order to focus only on the titles that may matter to the topic.

The keywords selection had been built up in three stages:

**Stage 1**: Building the keywords related to intervention (I)

The researcher conducted a search using the original search term for intervention and then reviewed the titles and abstracts of the results to identify any additional appropriate terms that were discovered. Once he felt confident that he had identified all of the main commonly used terms in order to avoid the risk of missing any studies.

**Stage 2**: Building the keywords related to Outcome (O)

In this stage, the keywords were extracted from the literature review and from the PIPs activities conducted in the CHIPPS pharmaceutical care plans.

The researcher searched using the original search term for intervention and reviewed the titles and abstracts to identify additional appropriate terms. Then, repeated the search until he was confident that he had identified all of the main commonly used terms.

**Stage 3**: Word exclusion from titles only

This stage had been conducted to give a more inclusive search. Once the researcher was satisfied with the search terms, he decided to review all the titles to identify any terms which would automatically indicate that the paper was inappropriate for the purposes of this study. These were used with the Boolean operand 'NOT' at the end of the search strategy.

With the refined strategy the researcher undertook the main scoping study. From all titles found, only 10% were reviewed. These were sampled by simple random sampling in which each of the titles had an equal and likely possibility to be selected in this sample, so, each title was marked by specific number and the selection of which depends on the possibility.

## 1.3.4. Inclusion and exclusion criteria

From the review of the titles, abstracts and papers inclusion and exclusion criteria were agreed between the researcher and primary supervisor according to the research question.

## 1.3.5. Development of data extraction tool

The data extraction tool was designed using Microsoft excel. This tool was guided by Cochrane Effective Practice and Organisation of Care (EPOC) review group data collection checklist to extract the following data from each identified study where possible (3):

- General characteristics of the included studies: title, author(s), year of study publication, study objective, design, setting, and country and characteristics of the participants: target population for the medication review intervention (e.g., training of HCPs or patients), and participants' sample size.

This tool had been tested in the scoping review to check its eligibility to be used in the main systematic review. So, after that the researcher decided to add the following points to the data extraction:

1. 'Who delivered the intervention' column to the general characteristics table.
2. The following data to be extracted as per the aim of the systematic review.

- All terms used to describe medication review interventions were conducted by HCPs in different settings.
- Description of medication review interventions.
- Description of processes used to develop definitions for medication review activities.

## 1.3.6. Quality assessment tool identification

To identify the most suitable quality assessment tool for the systematic review, the researcher collated the most used quality assessment tool. This step was conducted through preliminary search for the available quality assessment tool and the purpose for each of them aiming to select the most appropriate one for the systematic review.

## 1.4. Results

## 1.4.1. Database identified by scoping review

The initial search identified the systematic review that had been used in studies reporting or investigating the medication review intervention. The systematic reviews were reviewed to identify the databases used within them are shown in table 2 below.

**Table 2: Summary of the reviewed systematic reviews to identify the databases**

| **Title** | **Authors and year** | **Databases** | | | | | | | | | | |
|---|---|---|---|---|---|---|---|---|---|---|---|---|
| | | Medline | Embase | IPA | CINAHL | Cochrane | Biblioteca Virtual em Saúde (BVS) | PubMed | Scopus | Web of science | PsycInfo | Grey literature |
| Pharmacist-led medication review in community settings: An overview of systematic reviews | N. Jokanovic et al, 2017 (4) | √ | √ | √ | √ | √ | X | X | X | X | X | X |
| Pharmacist-participated medication review in different practice settings: Service or intervention? An overview of systematic reviews | De silva et al, 2019 (5) | X | √ | X | X | √ | √ | √ | √ | √ | X | X |
| Effectiveness of medication review: A systematic review and meta-analysis of randomised controlled trials | Victor Johan et al, 2017 (6) | √ | √ | X | X | X | X | X | X | √ | X | X |
| A systematic review and meta-analysis of pharmacist- | Ernieda Hatah et | √ | √ | √ | X | X | X | X | X | X | X | X |

| | | | | | | | | | | | | |
|---|---|---|---|---|---|---|---|---|---|---|---|---|
| led fee-for-services medication review | al, 2013 (7) | | | | | | | | | | | |
| Medication review and reconciliation with cooperation between pharmacist and general practitioner and the benefit for the patient: A systematic review | Marlies M et al, 2012 (8) | X | √ | X | X | X | X | √ | X | X | X | X |
| Residential aged care medication review to improve the quality of medication use: A systematic review | K. Thiruchelvam et al, 2017 (9) | X | X | √ | √ | √ | X | √ | X | X | X | X |
| Clinical medication review in Australia: A systematic review | N. Jokanovic et al, 2016 (10) | √ | √ | √ | √ | X | X | X | X | X | √ | X |
| Process, impact and outcomes of medication review in Australian residential aged care facilities: A systematic review | Esa Y. H. Chen et al, 2019 (11) | √ | √ | | √ | X | X | √ | X | X | X | √ |
| Effectiveness of pharmacist-led medication review in chronic pain management: Systematic review and meta-analysis | Hadi, Muhammad A. et al, 2014 (12) | √ | √ | √ | √ | X | X | X | X | X | √ | X |

The databases were evaluated for their appropriateness for use in this systematic review. The following databases were excluded from the previous systematic reviews:

- International Pharmaceutical Abstracts (IPA) (contain abstracts only)
- Cochrane Database (because the existing systematic reviews had been excluded from inclusion in this systematic review)
- Web of science (Web of Science contains only citations to journal publications published in international scientific indexing (ISI) - listed journals and excludes non-ISI journals)
- Biblioteca Virtual em Saúde (BVS) (not in English language)
- PubMed (contains the same information as Medline - search overlap)
- Scopus (It is currently limited to recent articles)
- Grey literature (contains studies that are not peer reviewed nor published)

However, the researcher decided to use the following databases for the systematic review:

- Embase (Ovid)
- Medline (Ovid)
- AMED (EBSCO)
- PsycInfo (EBSCO)
- CINAHL Complete (EBSCO)

## 1.4.2. Keyword selection according to Population, Intervention, Comparison and outcomes (PICO) framework

### Stage one for Intervention (I)

With the initial search for the intervention, we found 965 papers. On reviewing these we identified the additional term ‘Drug therapy review’. Adding this term increased our search to 1227 papers.

### Stage two for Outcome (O)

With the initial search for the outcomes, the researcher found 1129079 papers. On reviewing the pharmaceutical care plans the researcher identified the additional synonyms deprescribe, discontinue, hold, commence, initiate, switch, alter, step down, step up, test, train, educate, advise. Adding these synonyms increased the search to 5719922 papers.

### Stage three for exclusion of keywords from the titles only

In this stage the researcher combined keywords in stage one and stage two with excluded keywords, therefore, the number of hits was 7,635 papers. However, the researcher’s final decision is summarised in table 3.

**Table 3**: Summary of the main keywords identifies from original search and the additional words conducted in the reviewed search

| | Original search keywords | Additional words | Source |
|---|---|---|---|
| **Intervention (I)** | Medication review, drug utilisation review, drug review | Drug therapy review | Literature review |
| **Outcomes (O)** | stop, start, change, reduce, increase, monitor, educate, technical | ***Synonyms:*** deprescribe discontinue, hold, commence, initiate, switch, alter, step down, step up, test, train, advise | ***Original search keywords:*** literature review<br>***Synonyms:*** PCPs |
| **Excluded keywords (NOT)** | Evolution, guideline, survey, surveillance, biological, audit association, pharmacoepidemiology, physiological, stewardship, acupuncture, adjuvant, exercise, herbal, herb, fitness, epidemiologic, epidemiological, physiotherapy, chiropractic, complementary and alternative, complementary therapy, food and supplement, functional, intravenous, intrathecal, phytochemical, phytochemistry, radiation, teaching, radio*, rehabilitation, systematic review, child* | | |

## 1.4.3. Inclusion and exclusion criteria

Justification of inclusion and exclusion criteria are included in table 4 and 5 below.

**Table 4:** Inclusion criteria and the justification for their inclusion.

| Inclusion criteria | Justification |
|---|---|
| Studies that provided a description of medication review activities conducted by any health care professionals (HCPs) in different settings. | The researcher indicated that because medication review occurs throughout many settings.<br>To allow using this together with consensus methodology to develop a standardised international taxonomy for medication review interventions. |
| Medication review is based on patients with multi-morbidity | This inclusion will provide a general description of the medication review interventions. |
| Medication review conducted by any health care professional | Because medication review activities are carried out by a variety of health care professionals, including physicians, pharmacists, and nurses, this feature gives a consolidated picture of all medication review activities. |
| The papers must be written in English | To allow the literature to be read and understood by the research team (language limitation) |

**Table 5:** Exclusion criteria and the justification for their exclusion.

| Exclusion criteria | Justification |
|---|---|
| Studies are excluded if they are focused on medication reviews within a single disease state | Medication review interventions aimed at a single disease, such as cardiovascular disease, will provide specific information on that disease treatment. |
| The grey literature reviews will be excluded. | Grey literature reviews are not peer reviewed and indexed in a major bibliographic resource |
| Systematic review | If the systematic reviews were included, there is a risk of duplicate findings.<br>Unlikely to include data at the level required for extraction |
| Pilot studies | Unlikely to include data at the level required for extraction |
| Audits | Audits excluded because they are non-interventional activities |
| Posters and abstracts | excluded because they are unlikely to provide the level of detail required |
| Papers without empirical data such as protocols and abstracts only studies | Do not provide comprehensive information regarding the medication review intervention; hence, no medication review activities may be recorded within these resources. |

However, the researcher decided to include the following inclusion and exclusion criteria for the systematic review:

### Inclusion criteria

- Medication review studies that provided the nature and description of medication review activities.
- Medication reviews based on patients with multi-morbidity.
- Medication reviews conducted by any health care professional in different settings.

### Exclusion criteria

- Focused on medication reviews within a single disease state
- Systematic reviews
- Audits
- Posters
- Papers without empirical data such as protocols and abstracts only studies.
- Grey literature

### 1.4.4. Data extraction tool

According to the established inclusion and exclusion criteria, the data will be extracted as per general characteristics as in table 6.

**Table xx:** Summary of general characteristics of included studies

| Title | Author, year, and country | Objective | Design | Setting | Target population | Sample size |
|---|---|---|---|---|---|---|
| | | | | | | |

Then, this table had been piloted according to the extracted studies in this scoping review and the researcher decided to amend this table as per shown in table 7 below.

| Title | Author, year, and country | Objective | Design | Setting | Target population | Sample size | Who delivered the intervention |
|---|---|---|---|---|---|---|---|
| | | | | | | | |

Then, the data will be extracted as per the expected medication review activities terms as shown in table 8.

**Table xx:** terms used to describe in medication review activities

| Stop term | Start term | Dose increase term | Dose decrease term | Change term | Monitor term |
|---|---|---|---|---|---|
| | | | | | |

This tool had been piloted on the papers extracted from this scoping review and the researcher decided to amend the extraction tool as per shown in table 9.

| Terms used to describe in medication review activities | | | | | | | |
|---|---|---|---|---|---|---|---|
| Stop term | Start term | Dose increase term | Dose decrease term | Change term (for medication) | Change term (for formulation) | Change term (for timing) | Monitor term |

The other terms then separated in different table because we found in this review and expect to find more terms that are not classified.

### 1.4.5. Quality assessment tool identification

The most popular quality assessment tools used in medication review research are shown in table 10.

**Table 10:** quality assessment tools commonly used in medication review research

| Tool name | Purpose of the tool |
|---|---|
| Cochrane quality assessment tool (13) | Collaboration's tool for assessing risk of bias in randomised trials |
| AMSTAR (A MeaSurement Tool to Assess systematic Reviews) (14) | an instrument used in assessing the methodological quality of systematic reviews of RCTs. |
| CASP (Critical Appraisal Skills Programme) (15) | Randomised controlled trial standard checklist |
| COREQ (COnsolidated criteria for REporting Qualitative research) (16) | A checklist of items that should be included in reports of qualitative research. |
| JBI QARI Critical Appraisal tool (17) | Appraisal checklist for qualitative research. |
| Mixed Methods Appraisal Tool (MMAT) (18) | Quality assessment tools for mixed methods studies |
| Newcastle – Ottawa quality assessment tool (19) | To assess the quality of non - randomised **studies.** |
| ROBINS-I (Risk Of Bias In Non-randomized Studies Of Interventions) (20) | Tool for assessing risk of bias in non-randomised studies of interventions |

From the scoping review results, the researcher expected that the systematic review will encounter a range of different study designs. Therefore, the researcher selected the MMAT assessment tool to be applied for the systematic review. MMAT allows appraisal of the methodological quality of five types of studies: randomised controlled trials, non-randomised controlled trials, quantitative descriptive studies, qualitative studies and mixed method studies. The researcher looked at each included study and selected the appropriate type of study to appraise by responding to the screening questions with either 'Yes' or 'No' or 'Can't tell'. The higher the rating of the study, the higher its quality (18).

### 1.4.6. Prisma chart of scoping review results

This stage was conducted once the researcher identified the search terms and agreed inclusion and exclusion criteria according to PRISMA chart (21).

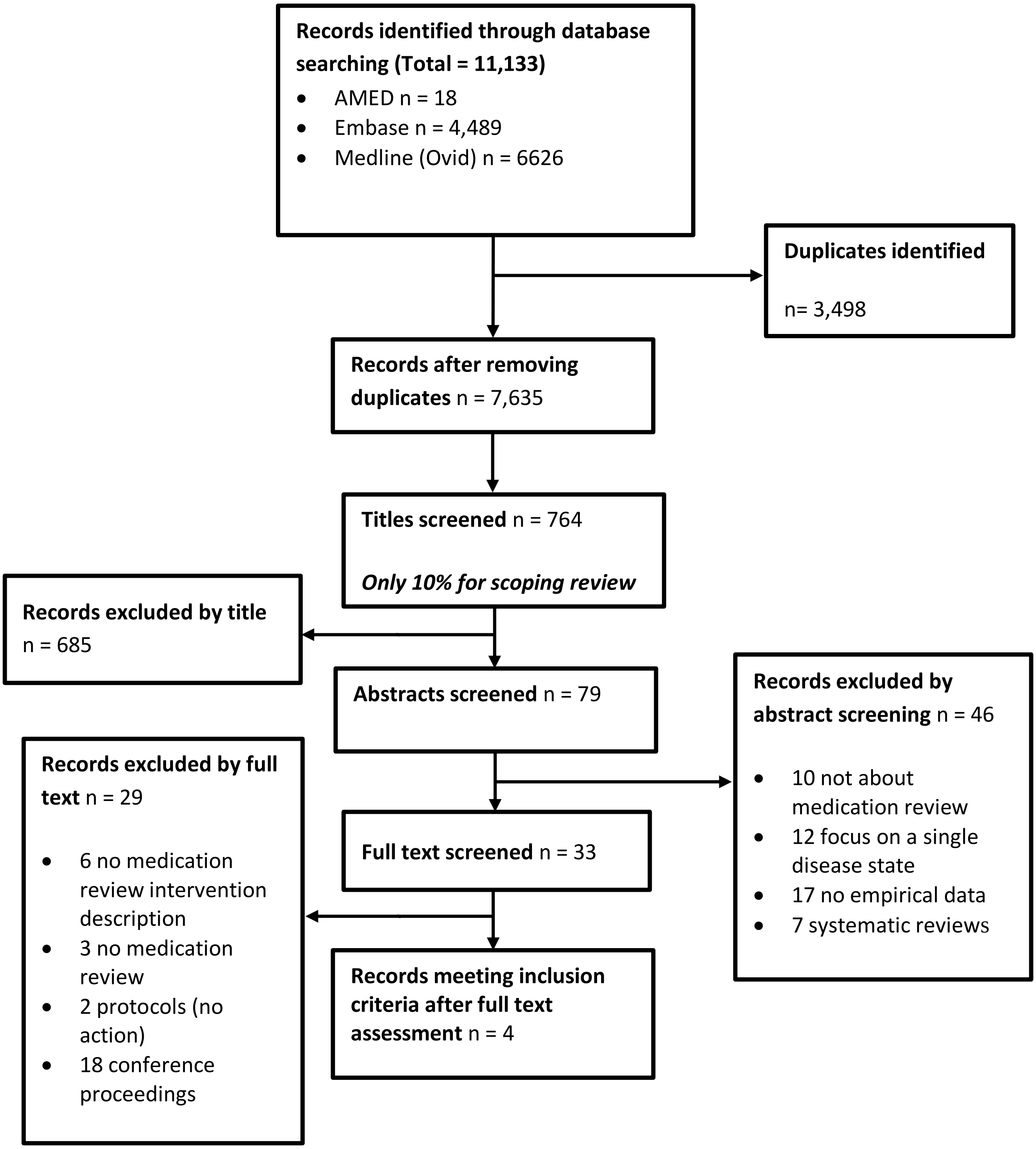


**Figure 1:** Prisma chart of the scoping review

**References**

1. Sayers A. Tips and tricks in performing a systemic review. Br J Gen Pract. 2008;58(547):136.
2. Sayers A. Tips and tricks in performing a systemic review. Br J Gen Pract. 2008;58(547):136.
3. Grimshaw J. Systematic reviews of the effectiveness of quality improvement strategies and programmes. Qual Saf Heal Care [Internet]. 2003 Aug 1;12(4):298–303. Available from: https://qualitysafety.bmj.com/lookup/doi/10.1136/qhc.12.4.298
4. Jokanovic N, Tan EC, Sudhakaran S, Kirkpatrick CM, Dooley MJ, Ryan-Atwood TE, et al. Pharmacist-led medication review in community settings: An overview of systematic reviews. Res Soc Adm Pharm [Internet]. 2017;13(4):661–85. Available from: http://dx.doi.org/10.1016/j.sapharm.2016.08.005
5. Silva R de OS, Macêdo LA, Santos GA dos, Aguiar PM, de Lyra DP. Pharmacist-participated medication review in different practice settings: Service or intervention? An overview of systematic reviews. Hills RK, editor. PLoS One [Internet]. 2019 Jan 10;14(1):e0210312. Available from: https://dx.plos.org/10.1371/journal.pone.0210312
6. Huiskes VJB, Burger DM, van den Ende CHM, van den Bemt BJF. Effectiveness of medication review: a systematic review and meta-analysis of randomized controlled trials. BMC Fam Pract [Internet]. 2017 Dec 17;18(1):5. Available from: http://bmcfampract.biomedcentral.com/articles/10.1186/s12875-016-0577-x
7. Hatah E, Braund R, Tordoff J, Duffull SB. A systematic review and meta-analysis of pharmacist-led fee-for-services medication review. Br J Clin Pharmacol [Internet]. 2014 Jan;77(1):102–15. Available from: http://doi.wiley.com/10.1111/bcp.12140
8. Geurts MME, Talsma J, Brouwers JRBJ, de Gier JJ. Medication review and reconciliation with cooperation between pharmacist and general practitioner and the benefit for the patient: a systematic review. Br J Clin Pharmacol [Internet]. 2012 Jul;74(1):16–33. Available from: http://doi.wiley.com/10.1111/j.1365-2125.2012.04178.x
9. Thiruchelvam K, Hasan SS, Wong PS, Kairuz T. Residential Aged Care Medication Review to Improve the Quality of Medication Use: A Systematic Review. J Am Med Dir Assoc [Internet]. 2017 Jan;18(1):87.e1-87.e14. Available from:

https://linkinghub.elsevier.com/retrieve/pii/S1525861016304820
10. Jokanovic N, Tan ECK, van den Bosch D, Kirkpatrick CM, Dooley MJ, Bell JS. Clinical medication review in Australia: A systematic review. Res Soc Adm Pharm [Internet]. 2016 May;12(3):384–418. Available from: https://linkinghub.elsevier.com/retrieve/pii/S1551741115001242
11. Chen EYH, Wang KN, Sluggett JK, Ilomäki J, Hilmer SN, Corlis M, et al. Process, impact and outcomes of medication review in Australian residential aged care facilities: A systematic review. Australas J Ageing [Internet]. 2019 Sep 8;38(S2):9–25. Available from: https://onlinelibrary.wiley.com/doi/abs/10.1111/ajag.12676
12. Hadi MA, Alldred DP, Briggs M, Munyombwe T, Closs SJ. Effectiveness of Pharmacist-led Medication Review in Chronic Pain Management. Clin J Pain [Internet]. 2014 Nov;30(11):1006–14. Available from: http://journals.lww.com/00002508-201411000-00010
13. Higgins JPT, Altman DG, Gotzsche PC, Juni P, Moher D, Oxman AD, et al. The Cochrane Collaboration's tool for assessing risk of bias in randomised trials. BMJ [Internet]. 2011 Oct 18;343(oct18 2):d5928–d5928. Available from: https://www.bmj.com/lookup/doi/10.1136/bmj.d5928
14. Shea BJ, Reeves BC, Wells G, Thuku M, Hamel C, Moran J, et al. AMSTAR 2: a critical appraisal tool for systematic reviews that include randomised or non-randomised studies of healthcare interventions, or both. BMJ [Internet]. 2017 Sep 21;j4008. Available from: https://www.bmj.com/lookup/doi/10.1136/bmj.j4008
15. Long HA, French DP, Brooks JM. Optimising the value of the critical appraisal skills programme (CASP) tool for quality appraisal in qualitative evidence synthesis. Res Methods Med Heal Sci [Internet]. 2020 Sep 6;1(1):31–42. Available from: http://journals.sagepub.com/doi/10.1177/2632084320947559
16. Tong A, Sainsbury P, Craig J. Consolidated criteria for reporting qualitative research (COREQ): a 32-item checklist for interviews and focus groups. Int J Qual Heal Care [Internet]. 2007 Sep 16;19(6):349–57. Available from: https://academic.oup.com/intqhc/article-lookup/doi/10.1093/intqhc/mzm042
17. Williams V, Boylan A-M, Nunan D. Critical appraisal of qualitative research: necessity, partialities and the issue of bias. BMJ Evidence-Based Med [Internet]. 2020 Feb;25(1):9–11. Available from: https://ebm.bmj.com/lookup/doi/10.1136/bmjebm-

2018-111132

18. Hong QN, Fàbregues S, Bartlett G, Boardman F, Cargo M, Dagenais P, et al. The Mixed Methods Appraisal Tool (MMAT) version 2018 for information professionals and researchers. Educ Inf [Internet]. 2018 Dec 18;34(4):285–91. Available from: https://www.medra.org/servlet/aliasResolver?alias=iospress&doi=10.3233/EFI-180221
19. Stang A. Critical evaluation of the Newcastle-Ottawa scale for the assessment of the quality of nonrandomized studies in meta-analyses. Eur J Epidemiol [Internet]. 2010 Sep 22;25(9):603–5. Available from: http://link.springer.com/10.1007/s10654-010-9491-z
20. Sterne JAC, Savović J, Page MJ, Elbers RG, Blencowe NS, Boutron I, et al. Cochrane; ROBINS-I_detailed_guidance. BMJ. 2019;366(October):1–53.
21. Moher D, Shamseer L, Clarke M, Ghersi D, Liberati A, Petticrew M, et al. Evaluation of ASTM Standard Test Method E 2177, 6 Retroreflectivity of Pavement Markings in a Condition of 7 Wetness. Syst Rev. 2015;(January):1–9.
