# Supplementary material for: Terms used to describe and define activities undertaken as a result of the medication review process: Do they require standardisation? A systematic review
Source: Int J Clin Pharm. 2022 Nov 21;45(2):304–19. doi: 10.1007/s11096-022-01494-5 (PMC10147810; doi:10.1007/s11096-022-01494-5)
Supplement: Supplementary file 2 — Supplementary file2 (PDF 122 KB) [file 11096_2022_1494_MOESM2_ESM.pdf]

Search strategy:

Studies identified through electronic search aiming to identify all the reports of published studies. The systematic review protocol developed and registered on the international database of prospectively registered systematic reviews (PROSPERO).

The following databases used for the systematic review search:

- Embase (Ovid)
- Medline (Ovid)
- AMED (EBSCO)
- PsycInfo (EBSCO)
- CINAHL Complete (EBSCO)

The search strategy for this systematic review included using Boolean operators such as 'AND'' and 'OR' for combining together the search terms, 'ADJ2' as a proximity operator and 'NOT' for word exclusion. In addition, the researcher used the truncations (*) and wild cards (?)

(''Medication adj2 review', OR ''drug utili?ation review' OR 'drug adj2 review' OR drug adj2 therapy review' OR 'medicine adj2 review' OR 'pharmaceutical adj2 review')

AND

Stop* OR discontinu* OR hold* or start* OR initiat* OR commenc* OR monitor* OR test OR reduc* OR decreas* OR alter* OR chang* OR switch* OR technical OR computeri?ed OR educat* OR advis* OR increas*

AND

NOT (ti only) (Evolution OR Guideline OR Survey OR Surveillance OR Biological OR Audit OR Association OR Pharmacoepidemiology OR Physiological OR Stewardship OR Acupuncture OR Adjuvant OR Exercise OR Herbal OR Herb OR Fitness OR Epidemiologic OR Epidemiological OR Physiotherapy OR Chiropractic, Complementary and alternative, Complementary therapy, Food and supplement, Functional, Intravenous, Intrathecal, Phytochemical, Phytochemistry OR Radiation OR Teaching OR Radio* OR Rehabilitation OR Systematic review OR Child*).

Search results limited by two filters: publication type (journal) and the English language.

Table 1: Example of search strategy development through Medline

	Search	Results
1	(''medication review'' OR ''drug utilisation review'').mp. [mp=title, abstract, original title, name of substance word, subject heading word, floating sub-heading word, keyword heading word, organism supplementary concept word, protocol supplementary concept word, rare disease supplementary concept word, unique identifier, synonyms]	965
2	(''medication review'' OR ''drug utilisation review'' OR ''drug review'' OR ''drug therapy review'').mp. [mp=title, abstract, original title, name of substance word, subject heading word, floating sub-heading word, keyword heading word, organism supplementary concept word, protocol supplementary concept word, rare disease supplementary concept word, unique identifier, synonyms]	1227
3	(''Medication adj2 review', OR ''drug utili?ation review' OR 'drug adj2 review' OR drug adj2 therapy review' OR 'medicine adj2 review' OR 'pharmaceutical adj2 review').mp. [mp=title, abstract, original title, name of substance word, subject heading word, floating sub-heading word, keyword heading word, organism supplementary concept word, protocol supplementary concept word, rare disease supplementary concept word, unique identifier, synonyms]	4956
4	(stop OR start OR test OR decrease OR increase OR education OR technical OR alter).mp. [mp=title, abstract, original title, name of substance word, subject heading word, floating sub-heading word, keyword heading word, organism supplementary concept word, protocol supplementary concept word, rare disease supplementary concept word, unique identifier, synonyms]	4575442
5	(''Medication adj2 review' OR ''drug utili?ation review' OR 'drug adj2 review' OR 'drug adj2 therapy review' OR 'medicine adj2 review' OR 'pharmaceutical adj2 review').mp. [mp=title, abstract, original title, name of substance word, subject heading word, floating sub-heading word, keyword heading word, organism supplementary concept word, protocol supplementary concept word, rare disease supplementary concept word, unique identifier, synonyms]	5180
6	(stop* OR discontinu* OR hold* OR start* OR initiat* commenc* OR monitor* OR test OR reduc* OR decreas* OR alter* OR chang* OR switch* OR technical OR computeri?ed OR educat* OR advis* OR train* OR increas*).mp. [mp=title, abstract, original title, name of substance word, subject heading word, floating sub-heading word, keyword heading word, organism supplementary concept word, protocol supplementary concept word, rare disease supplementary concept word, unique identifier, synonyms]	1129076
7	5 AND 6 AND NOT (ti only) (Evolution OR Guideline OR Survey OR Surveillance OR Biological OR Audit OR Association OR Pharmacoepidemiology OR Physiological OR Stewardship OR Acupuncture OR Adjuvant OR Exercise OR Herbal OR Herb OR Fitness OR Epidemiologic OR Epidemiological OR Physiotherapy OR Chiropractic, Complementary and alternative, Complementary therapy, Food and supplement, Functional, Intravenous, Intrathecal, Phytochemical, Phytochemistry OR Radiation OR Teaching OR Radio* OR Rehabilitation OR Systematic review OR Child*)	6719
