# Supplementary material for: Terms used to describe and define activities undertaken as a result of the medication review process: Do they require standardisation? A systematic review
Source: Int J Clin Pharm. 2022 Nov 21;45(2):304–19. doi: 10.1007/s11096-022-01494-5 (PMC10147810; doi:10.1007/s11096-022-01494-5)
Supplement: Supplementary file 3 — Supplementary file3 (PDF 148 KB) [file 11096_2022_1494_MOESM3_ESM.pdf]

Table 1: Summary of quality assessment for Quantitative non-randomised studies

**Author and year**	Clear research question	Data addressed research questions	Representative	Appropriate outcome	Outcome data complete	Confounders considered	Intervention as intended	**Total**
Alldred et al. (2007) [1]	Yes	Yes	Yes	Yes	Yes	No	Yes	6
Chau et al. (2015) [2]	Yes	Yes	Yes	Yes	Yes	No	Yes	6
Krska et al. (2006) [3]	Yes	Yes	Yes	Yes	Yes	No	Can't tell	5
Kwint et al (2012) [4]	Yes	Yes	Yes	Yes	Yes	No	Yes	6
Kwint et al. (2013) [5]	Yes	Yes	Yes	Yes	Yes	No	Yes	6
Laaksonen (2010) [6]	Yes	Yes	Yes	Yes	Yes	No	Yes	6
Petty et al. (2002) [7]	Yes	Yes	Yes	Yes	Yes	No	Can't tell	5
Lenander et al. (2018) [8]	Yes	Yes	Yes	Yes	Yes	No	Yes	6

Table 2: Summary of quality assessment for quantitative descriptive studies

**Author and year**	Clear research question	Data addressed research questions	Relevant sampling	Representative	Appropriate measurements	Low bias risk	Appropriate statistical analysis	Total
Fog et al. (2017) [9]	Yes	Yes	Yes	Yes	Yes	Yes	Yes	7
Christopher et al. (2012) [10]	Yes	Yes	Yes	Yes	Yes	Yes	Yes	7
Modig et al. (2015) [11]	Yes	Yes	Yes	Yes	Yes	Yes	Yes	7

Smith et al. (2002) [12]	Yes	Yes	Yes	Yes	Yes	Yes	Yes	7
Chan et al. (2018) [13]	Yes	Yes	Yes	Yes	Yes	Yes	Yes	7

Table 3: Summary of quality assessment for quantitative randomised controlled trials

**Author and year**	Clear research question	Data addressed research questions	Randomised appropriately	Groups compared at baseline	Outcome data complete	assessors blinded	Participants adhere to intervention	**Total**
Hessleman et al. (2015) [14]	Yes	Yes	Yes	Yes	Yes	No	Yes	6
Vinks et al. (2009) [15]	Yes	Yes	Yes	Yes	Yes	No	Yes	6
Kwint et al. (2011) [16]	Yes	Yes	Yes	Yes	Yes	No	Yes	6
Zermansky et al. (2006) [17]	Yes	Yes	Yes	Yes	Yes	Can't tell	Yes	6
Al Alaweneh et al. (2018) [18]	Yes	Yes	Yes	Yes	Yes	No	Yes	6
Elliott et al. (2012) [19]	Yes	Yes	Yes	Yes	Yes	No	Yes	6
Sorensen et al. (2004) [20]	Yes	Yes	Yes	Yes	No	No	Yes	5
Zermansky et al (2001) [21]	Yes	Yes	Yes	Yes	Yes	No	Yes	6

References

1. Alldred DP, Zermansky AG, Petty DR, Raynor DK, Freemantle N, Eastaugh J, et al. Clinical medication review by a pharmacist of elderly people living in care homes: pharmacist interventions. Int J Pharm Pract [Internet]. 2010 Feb 18;15(2):93–9. Available from: https://academic.oup.com/ijpp/article/15/2/93/6137109
2. Chau SH, Jansen APD, van de Ven PM, Hoogland P, Elders PJM, Hugtenburg JG. Clinical medication reviews in elderly patients with polypharmacy: a cross-sectional study on drug-related problems in the Netherlands. Int J Clin Pharm. 2016;38(1):46–53.
3. Krska J, Gill D, Hansford D. Pharmacist-supported medication review training for general practitioners: feasibility and acceptability. Med Educ [Internet]. 2006 Dec;40(12):1217–25. Available from: http://doi.wiley.com/10.1111/j.1365-2929.2006.02633.x
4. Kwint HF, Faber A, Gussekloo J, Bouvy ML. The contribution of patient interviews to the identification of drug-related problems in home medication review. J Clin Pharm Ther [Internet]. 2012 Dec;37(6):674–80. Available from: http://doi.wiley.com/10.1111/j.1365-2710.2012.01370.x
5. Kwint HF, Faber A, Gussekloo J, Bouvy ML. Completeness of medication reviews provided by community pharmacists. J Clin Pharm Ther [Internet]. 2014 Jun;39(3):248–52. Available from: http://doi.wiley.com/10.1111/jcpt.12132
6. Laaksonen R, Duggan C, Bates I. Performance of Community Pharmacists in providing clinical medication reviews. Ann Pharmacother. 2010;44(7–8):1181–90.
7. Petty DR, Zermansky AG, Raynor DK, Lowe CJ, Freemantle N, Vail A. Clinical medication review by a pharmacist of elderly patients on repeat medications in general practice - Pharmacist interventions and review outcomes. Int J Pharm Pract. 2002;10(1):39–45.
8. Lenander C, Bondesson Å, Viberg N, Beckman A, Midlöv P. Effects of medication reviews on use of potentially inappropriate medications in elderly patients; a cross-sectional study in Swedish primary care. BMC Health Serv Res. 2018;18(1):1–9.
9. Fog AF, Kvalvaag G, Engedal K, Straand J. Drug-related problems and changes in drug utilization after medication reviews in nursing homes in Oslo, Norway. Scand J Prim Health Care [Internet]. 2017;35(4):329–35. Available from: https://doi.org/10.1080/02813432.2017.1397246
10. Freeman CR, Cottrell WN, Kyle G, Williams ID, Nissen L. An evaluation of medication review reports across different settings. Int J Clin Pharm. 2013;35(1):5–13.
11. Modig S, Holmdahl L, Bondesson Å. Medication reviews in primary care in Sweden: importance of clinical pharmacists' recommendations on drug-related problems. Int J Clin Pharm. 2016;38(1):41–5.
12. Smith MA, Simpson JM, Benrimoj SI. General practitioner acceptance of medication review in Sydney nursing homes. J Pharm Pract Res. 2002;32(3):227–31.
13. Chan WWT, Dahri K, Partovi N, Egan G, Yousefi V. Evaluation of collaborative medication reviews for high-risk older adults. Can J Hosp Pharm. 2018;71(6):356–63.
14. Heselmans A, van Krieken J, Cootjans S, Nagels K, Filliers D, Dillen K, et al. Medication review by a clinical pharmacist at the transfer point from ICU to ward: a randomized controlled trial. J Clin Pharm Ther [Internet]. 2015 Oct;40(5):578–83. Available from: http://doi.wiley.com/10.1111/jcpt.12314
15. Vinks THAM, Egberts TCG, De Lange TM, De Koning FHP. Pharmacist-based medication review reduces potential drug-related problems in the elderly: The SMOG controlled trial. Drugs and Aging. 2009;26(2):123–33.
16. Kwint HF, Faber A, Gussekloo J, Bouvy ML. Effects of medication review on drug-related problems in patients using automated drug-dispensing

systems: A pragmatic randomized controlled study. Drugs and Aging. 2011;28(4):305–14.
17. Zermansky AG, Alldred DP, Petty DR, Raynor DK, Freemantle N, Eastaugh J, et al. Clinical medication review by a pharmacist of elderly people living in care homes—randomised controlled trial. Age Ageing [Internet]. 2006 Nov 1;35(6):586–91. Available from: http://academic.oup.com/ageing/article/35/6/586/14651/Clinical-medication-review-by-a-pharmacist-of
18. Al alawneh M, Nuaimi N, Basheti IA. Pharmacists in humanitarian crisis settings: Assessing the impact of pharmacist-delivered home medication management review service to Syrian refugees in Jordan. Res Soc Adm Pharm [Internet]. 2019;15(2):164–72. Available from: https://doi.org/10.1016/j.sapharm.2018.04.008
19. Elliott RA, Martinac G, Campbell S, Thorn J, Woodward. MC. Pharmacist-led medication review to identify medication-related problems in older people referred to an Aged Care Assessment Team: A randomized comparative study. Drugs and Aging. 2012;29(7):593–605.
20. Sorensen L, Stokes JA, Purdie DM, Woodward M, Elliott R, Roberts MS. Medication reviews in the community: Results of a randomized, controlled effectiveness trial. Br J Clin Pharmacol. 2004;58(6):648–64.
21. Zermansky AG, Petty DR, Raynor DK, Freemantle N, Vail A, Lowe CJ. Primary care prescriptions in general practice. Bmj. 2001;323(December):1340–3.
